# Air Quality in a Dental Clinic during Er:YAG Laser Usage for Cavity Preparation on Human Teeth—An Ex-Vivo Study

**DOI:** 10.3390/ijerph182010920

**Published:** 2021-10-17

**Authors:** Angeliki Karveli, Ioannis G. Tzoutzas, Panagiotis Ioannis Raptis, Emmanouil-George C. Tzanakakis, Eleftherios Terry R. Farmakis, Constantinos G. Helmis

**Affiliations:** 1 Private Practice, 12134 Athens, Greece; angelikikarveli@hotmail.com; 2 Department of Operative Dentistry, School of Dentistry, National and Kapodistrian University of Athens, 11527 Athens, Greece; tzoudent@dent.uoa.gr (I.G.T.); tzanakak@dent.uoa.gr (E.-G.C.T.); 3 Department of Physics, University of Athens, and National Observatory, 11527 Athens, Greece; piraptis@noa.gr; 4 Department of Endodontics, School of Dentistry, National and Kapodistrian University of Athens, 11527 Athens, Greece; 5 Division of Applied Physics, Department of Physics, University of Athens, 11527 Athens, Greece; chelmis@phys.uoa.gr

**Keywords:** minimally invasive dentistry, laser, Er:YAG, air quality, Volatile Organic Compounds (VOCs), particulate matter, PM10, PM2.5, working environment

## Abstract

Chemical air pollution in dental clinics consists of the emission of gases and particulate matter (PM), both generated by dental equipment and tooth tissues. One basic application of Erbium Laser devices is cavity preparation on human teeth due to its strong affinity to water and hydroxyapatite. The objective of this study was the evaluation of indoor air quality during the application of an Er:YAG laser, as a dentin removal instrument, in a Dental Clinic. Particulate Matter (PM) was measured using the standard method of EN legislation. In order to measure total Volatile Organic compounds (VOCs), a portable monitor was used. In the first experiment, PM10 and PM2.5 concentrations were increased by approximately 10 and 15 times, respectively. From the second experiment it can be concluded that neither of the measured particle concentrations exceeded the recommended indoor limit values while windows were open, although laser influence was still detectable. Within the limitations applied herein, it was found that Er:YAG laser activity for hard dental tissue removal was associated with high PM and TVOCs concentration values in the working environment, under insufficient or no ventilation. Physical ventilation in the aforementioned setting proved to be an important key factor in improving air quality, as both PM and TVOCs concentrations decreased significantly.

## 1. Introduction

During the last decades, and more importantly nowadays, in the COVID-19 era, the scientific community has been increasingly interested in the air quality at indoor spaces and its effects on human health [1]. Specific considerations have been raised for dental clinics, due to airborne microbial and chemical air pollutants [2]. Microbial air pollution is related to biological infectious agents [3,4] that can be transmitted via aerosols to patients or dental professionals and has been extensively studied by many researchers [5,6,7]. Also, investigation has been carried out with respect to the chemical components [8,9]. Chemical air pollution in dental clinics consists of the emission of gases and particulate matter (PM), both generated by dental equipment and tooth tissues.

Some of the gas phase contaminants generated and emitted from various dental procedures are the Volatile Organic Compounds (VOCs). These are organic chemicals that have a high vaporization at ordinary room temperature due to their low boiling point. This causes large numbers of molecules to evaporate or sublimate from the liquid or solid form of the compound and diffuse into the surrounding air. These VOCs commonly include methanol, formaldehyde, methyl-acrylate, methyl-methacrylate and isobutyl-methacylate [10,11]. Adverse health symptoms associated with high VOC concentrations mostly include irritant effects due to mucous membrane infection, but also systemic effects such as fatigue and difficulty concentrating. Prolonged exposure may cause allergies, liver or kidney damage, asthma syndrome and various toxic effects [5,12]. In many publications dealing with VOCs, a tendency can be observed not to report the concentrations of all analyzed VOCs individually, but rather indicate the total concentration of VOCs under the term “Total Volatile Organic Compounds” (TVOCs). One of the reasons is that the interpretation of one single parameter is simpler and faster than the interpretation of the concentrations of several dozens of VOCs typically detected indoors.

Indoor particulate matter (PM) with a diameter of less than 50 μm, may be invisible to the naked eye but can sustain as aerosols for prolong periods of time [13]. The major components of PM are sulfate, nitrates, ammonia, sodium chloride, black carbon, mineral dust and water. In other terms, it is a complex mixture of solid and liquid particles, of organic and inorganic substances, suspended in the air. These particles have been associated with various effects on human health but mainly with respiratory disorders. Penetration in the respiratory tract and the ability of the respiratory system to remove the undesirable invaders depend on the particle size and chemical composition. Coarse (2.5–10 μm), fine (0.1–2.5 μm) and ultrafine (<0.1 μm) PM tend to deposit in diverse regions of the respiratory system [12]. The smaller the particles, the deeper they penetrate into the lungs. Several studies have shown a positive correlation between indoor PM2.5 (<2.5 μm) and the presence of bronchitis and asthmatic symptoms [14].

Larger particles tend to be trapped in the nose, mouth or throat. For this reason, PM10 are referred to as inhalable particulate matter and are considered as an indoor air quality indicator [15]. On the other hand, PM2.5 are referred to as respirable and can enter the alveolar sac [15]. Chronic exposure to particles, contributes to the risk of developing cardiovascular and respiratory diseases, as well as of lung cancer. Most dental aerosols have a diameter of 5 μm or less and therefore long-term exposure is potentially hazardous not only for patients, but mainly for the dental staff [2,16,17]. Today there are quite a few methodologies to quantify air contamination in Dental Clinics [15].

Apart from the high-speed air turbine, which has been a known cause of airborne contamination for decades, modern Dentistry utilizes another technology that also may add to the deterioration of the dental office air quality: Laser devices, including the Erbium family ones [18].

The main advantage of Er lasers (either Er:YAG, or Er:Cr:YSGG) is that they can remove composite resin materials from dental restorations with minimum thermal stress to the dental pulp [19,20]. The specific wavelength (2940 nm), due to its strong affinity to water and hydroxyapatite (absorption), causes explosive vaporization of the internal water, mineral removal and results in a porous dental surface [21,22]. This ablating mechanism is expected to create airborne particulate matter. Additionally, any Dentist who has applied (in vitro or in vivo) Er Laser irradiation for restoration purposes, (cavity preparation, or material removal) can recall the distinct odor in the working environment.

Thus, the objective of this study was the evaluation of indoor air quality during the application of Er:YAG laser, as a dentin removal instrument, in a Dental Clinic. The present experimental study aimed at evaluating the air quality of an office room with similar dimensions to a typical solo dental practice, as well as in a multi-dental-unit clinic, when using Er:YAG lasers for cavity preparations on human teeth, in conjunction with different air-ventilation conditions. Thus, the null hypothesis supported that there would be an increase in TVOCs, PM10 and PM2.5 values, due to the operation of the laser device, regardless of the environmental conditions.

## 2. Materials and Methods

For the aforementioned evaluation, two experiments were planned and performed in the Dental School, National and Kapodistrian University of Athens, Greece. In both experiments, tooth cavities were prepared on extracted teeth, using an Er:YAG laser, while the same measuring instruments were used in order to assess the indoor concentration of PM and total VOCs. For both experiments, an Er:YAG laser device (Smart 2940 D Plus, DEKA, Calenzano, Italy) was used. The laser was set at 20 Hz frequency, and a pulse of 420 mJ (when working on enamel) or 200 mJ (when working on dentin), respectively, and water setting was at 100%, according to manufacturer’s instructions.

A quartz fiber tip of a 1-mm diameter was used in a close-contact mode (10 mm from the irradiated surface). The teeth used (11 in total) were extracted impacted third molars, with patients’ ages ranging between 25–34 years old [23]. Informed consent was released by all patients and the protocol was approved by the Ethics Committee of the Dental School of the National and Kapodistrian University of Athens, Greece (REF. 233b/10.04/2014).

External deposits and tissue remnants were removed from the teeth by placing them in 2.5% NaOCl solution for 5 min and the remains were removed with curettes. Specimens were stored in sterile saline (0.9% NaCl) at 4 °C until use. During cavity creation, each tooth was hand-held and each cavity was completed in three sessions. Each session focused on one layer at a time and lasted 1 min. Small intermissions of approximately thirty seconds were taken between sessions for dental tissue thermal relaxation. No Dental Unit High Vacuum Suction was used near the operating field, neither central room ventilation was operated.

### 2.1. Experiment 1

The first experiment took place in an office with dimensions similar to a typical operating room in a private dental clinic according to the local regulations (Greek Ministeral Decree 84/2001). The floor of the office was 9 m^2^ and 3 m in height (27 m^3^). The recordings lasted four days. During that time, there was no physical or technical ventilation and the door opened only a few times. A person was working on a PC inside the room for four hours on Day 0. Recordings for background ambient conditions were made on Day 0 (Baseline recordings), on Day 1 the access was restricted to all, so, no activity took place in the room and on Day 2, the Er:YAG laser device was used on two extracted teeth. No other dental procedures took place in the room.

### 2.2. Experiment 2

The second experiment took place at the Postgraduate Dental Clinic, Endodontics, Operative Dentistry and Periodontology at the School of Dentistry, University of Athens, over a 20-day span. The Clinic extended over an area of 150 m^2^ and was 3 m in height, guesting or housing 15 working dental units in it. It operated in two shifts, one between 08:00–12:30 and the other 13:00–17:00. During the experimental period there were 10–30 persons present inside the clinic. The Er:YAG laser apparatus was used on three occasions (on Day 11, Day 16 and Day 18). Since the environment in the clinic represents an everyday dental practice, different kinds of dental treatment were provided at the same time as the experiment, under various non-standardized conditions. While the measurements were being performed, on some days the windows were open and on some they were closed, and there was a maximum of 30 people working inside. During the operating hours of the clinic there were 20 people on average. Room ventilation conditions varied according to the amount (number) of open windows, which should be considered, and the ventilating surface ranged from 7–13 m^2^. The Er:YAG laser device was used on three teeth on each of these three days. Cavity preparation (Class II preparation) at each tooth lasted approximately 10 min treating both enamel and dentin in all cases. On each of these occasions, 3 teeth were used, while each tooth underwent laser processing for 3 one-minute intervals, until the desired cavity was achieved. During the rest of the days, measurements were taken in order to assess the levels of the PM and total VOCs background concentrations in the clinic. However, at the same time, various dental works took place in the clinic during post-graduate student training. Consequently, these measurements constitute a background picture only with respect to the Er:YAG laser usage.

### 2.3. Instrumentation and Experimental Set Up

Particulate Matter (PM) was measured using the standard method of EN legislation described at PrEN12341-2012-07. This method uses gravimetric measurements for the determination of the PM10 or PM2.5 mass concentration. Two air pumps (224-PCMTX8, SKC, PA, USA) with a flow rate of 2.3 m^3^/h were used simultaneously for 24 h periods. The pumps collected samples on quartz filters with diameters of 47 mm. Samples were conditioned before and after use in a controlled room with steady conditions (T = 20°, RH = 50–55%). Each sample was weighted every 24 h, at least two times, values with more than 10% relative difference were ignored and the mean value was used. Weighting was performed using a KERN 770-13 balance (Kern &Sohn GmbH, Ettenheim, Germany) reaching an accuracy of 0.01 mg. In order to measure total VOCs, a portable monitor was used (1 ppb resolution) (Rae Systems PpbRAE, San Jose, CA, USA). The instrument has a photoionization detector (PID) with a gas-discharge lamp of 9.8 eV, 10.6 eV or 11.7 eV. Recording frequency was 1 measurement per minute and the accuracy of the instrument should be considered to be about 10%. The measured values of TVOCs are isobutylene equivalent, and conversion from ppb to μg/m^3^ has been done by multiplying the measured value with the factor 2.3, according to a previous report [24].

## 3. Results

### 3.1. Experiment1 (Baseline Measurements)

#### 3.1.1. Particulate Matter

PM2.5 and PM10 background concentration values measured on Day 0 and those measured on Day 2 when the laser was in use are presented on Table 1. PM10 and PM2.5 concentrations on Day 2 increased by approximately 10 and 15 times, respectively. Small room dimensions, without ventilation, maintained particle concentrations at very high levels for a long period of time. The most alarming aspect with respect to potential health risks is the fact that combustion of organic matter caused extremely high concentration of fine particles (especially for PM2.5), which are more respiratory (~10 times the limit value) (Table 1).

#### 3.1.2. Total VOCs

**Figure 1:**
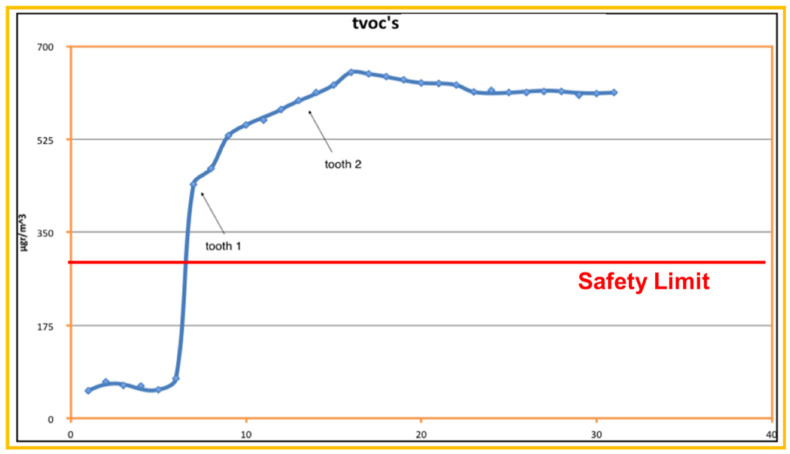
Detailed presentation of TVOC’s recordings (over a 40 min span), on D2 for the period of Er:YAG Laser usage, 10:35 to 10:55. The arrows indicate the completion of working time for each tooth.

**Figure 2:**
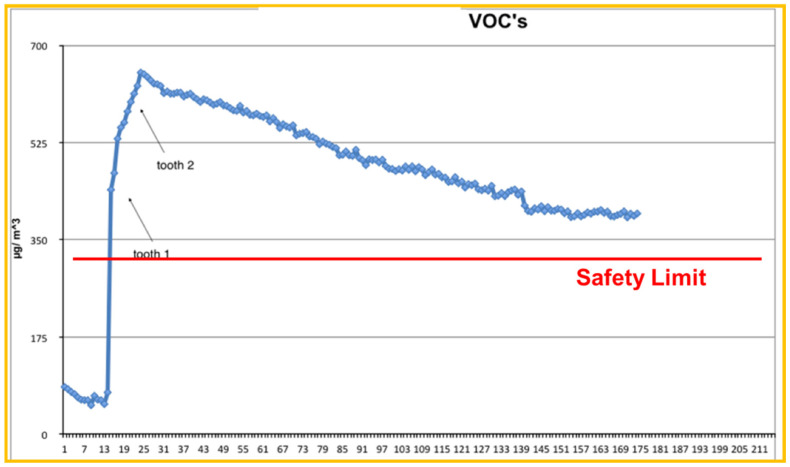
Presentation of TVOCs’ recordings (over a 3-h span), on D2 for a period of 20 min working with Er:YAG Laser usage. The arrows indicate the completion of working time for each tooth.

TVOCs’ concentration distribution on Day 2 is presented in Figure 1 and Figure 2.

Figure 1 focuses on the first thirty minutes within which Er:YAG laser activity took place. Measured TVOCs’ concentration values just before the beginning of dental work are considered as background values (~70 μg/m^3^). These values increased continuously for about 10 min, overcoming the safety limits (300 μg/m^3^) and reaching the maximum value of 645 μg/m^3^ (10 times greater than background values). It is important to notice that concentration values rose at a high rate upon the initiation of laser’s application. This clearly demonstrates the Er:YAG laser’s effects on TVOCs’ concentration. Figure 2 shows a wider picture of the same day’s concentration distribution, since concentration values are presented up to 2.5 h after the Er:YAG laser’s application. Additionally seen in this figure is the very low rate of concentration decrease. TVOCs’ concentration was sustained above recommended safety limits for more than 2 h after the completion of the Er:YAG laser’s activity. So, in cases with no ventilation, one should also consider the very slow rest-off time for TVOCs’ concentrations.

### 3.2. Experiment 2

#### 3.2.1. Particulate Matter

**Figure 3:**
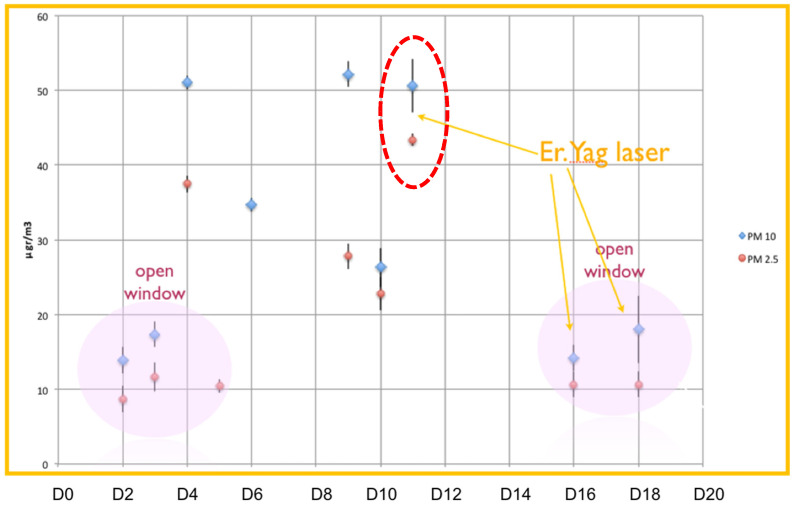
PM10 and PM 2.5 24 h value for Experiment 2. On Days D2, D3, D5, D16 and D18 windows were open, and on all other days closed. Er:YAG Laser operated on Days D11, D16 and D18.

Recorded values of PM2.5 and PM10 concentrations during the first experiment are presented in Table 2 (in mean values), and in Figure 3 (in detail).

In the same figure, one can distinguish the days when clinic’s windows were open (Days D2, D3, D5, D16 and D18), as well as the days when the Er:YAG laser was used (Days D11, D16, D18). When windows were open, both PM2.5 and PM10 concentration values were the lowest recorded within the experimental period, closely resembling the outdoor ones. On these days, PM2.5 concentration was approximately 10 μg/m^3^ and PM10 concentration ranged between 14 and 19 μg/m^3^. Neither of the measured particle’s concentrations exceeded the recommended indoor limit values while windows were open. Among values for periods with open windows. a small increase is indicated, especially for PM10 when the Er:YAG laser was used.

When windows were closed, PM2.5 and PM10 concentration values ranged between 23–43 μg/m^3^ and 27–52 μg/m^3^, respectively (with maximum indoors acceptable values 25 and 50 μg/m^3^ for PM2.5 and PM10 respectively). PM concentration was increased up to four times, exceeding in some cases safety threshold values. The great variation among these daily values is attributed to diverse dental activity in the clinic. However, D11 stood out for high concentrations concerning both PM2.5 and PM10, an event associated with the Er:YAG laser utilization in the Clinic. This was the day when PM2.5 concentration reached its maximum value (43 μg/m^3^), which exceeded safety limits, while PM10 concentration was at 50 μg/m^3^, similar to other days with no ventilation. Indoor air quality appears to be downgraded (negatively affected) due to laser activity, with or without room ventilation.

#### 3.2.2. Total VOCs

**Figure 4:**
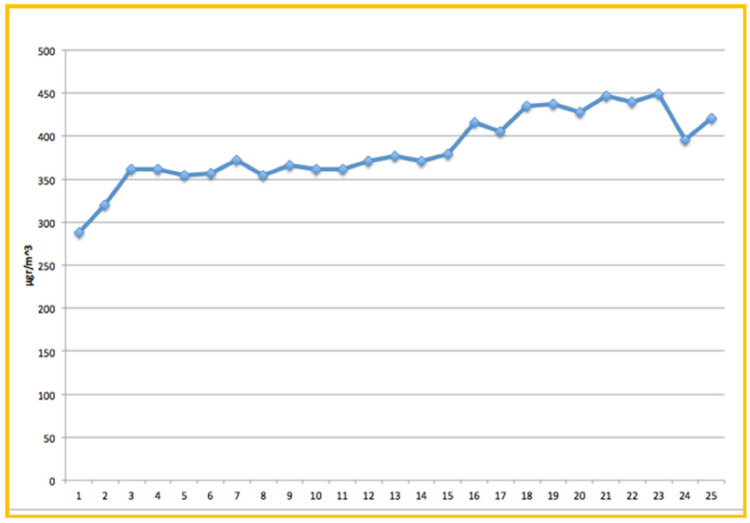
Background levels of TVOCs, during the operation of the dental Clinic without any laser activity, and no ventilation. The negative impact of no-ventilation during dental activity on the air quality, is obvious (safety limit is considered at 300 μg/m^3^).

During the second experiment, TVOCs’ concentration measurements were taken on four non-consecutive days. On Day 0 (D0), measured concentration values were considered as background levels (no laser use) and they marginally exceed the suggested TVOCs limit values, as they vary between 300–450 μg/m^3^ (Figure 4).

**Figure 5:**
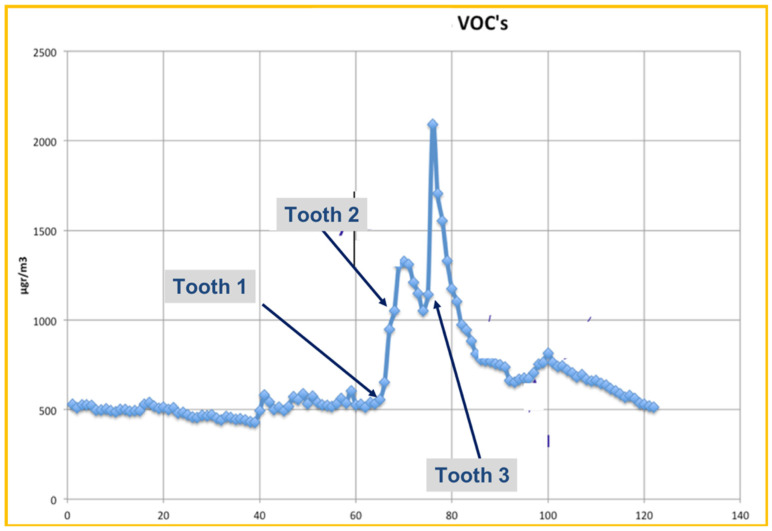
Recordings (over a 2-h span) of TVOCs on D11, during Laser operation. The arrows indicate the initiation of the laser device for cavity preparation, for each of the three teeth. No ventilation was taking place on that day. Take note of the hazardous values recorded.

**Figure 6:**
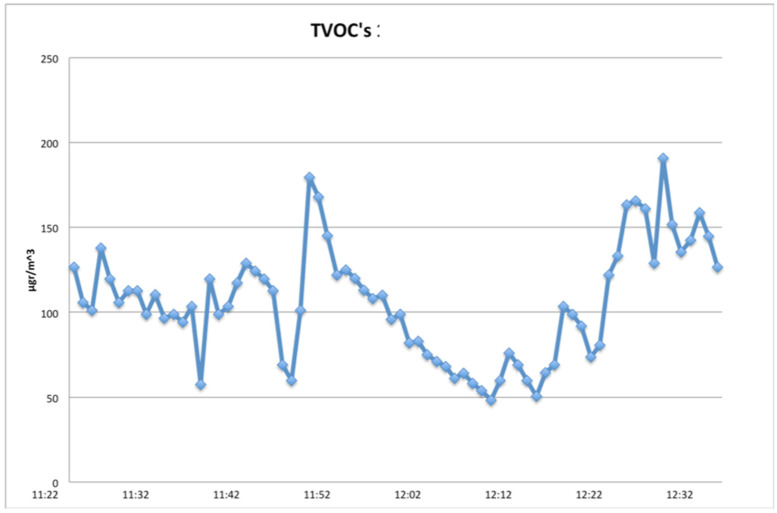
TVOCs on D16. Windows were open and Er YAG Laser was in use from 11:50 to 12:20 (1-h recording).

**Figure 7:**
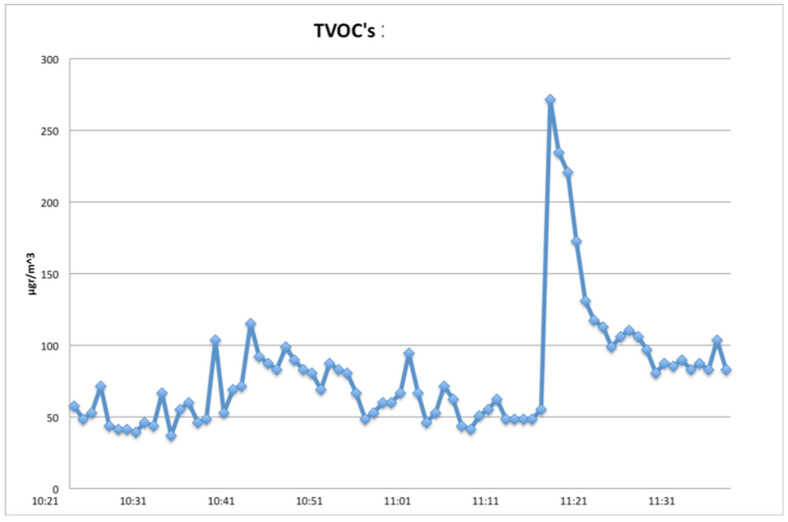
TVOCs on D18. Windows were open and Er:YAG Laser was in use from 11:10 to 11:30 (1-h recording).

The other three days were Days D11, D16 and D18, when tooth cavities were created using the Er:YAG laser, additionally to other dental procedures taking place in the clinic. The resulting VOCs’ concentration distribution on these days are presented in Figure 5, Figure 6 and Figure 7, respectively.

Measurements on D11 covered a 2-h time period and all windows were closed at the time (Figure 5). During the first hour, TVOCs’ concentration values varied between 450 μg/m^3^ and 600 μg/m^3^. These levels were already above the recommended values and this can be attributed to the other dental activities taking place in the clinic in combination with low ventilation. A detailed description of basic dental works’ influence on TVOC’s has been described in the literature [1].

At 12:20 pm, a sharp concentration increase began, reaching two consecutive maximum values of 1300 μg/m^3^ and 2100 μg/m^3^. At that time, the Er:YAG laser was first applied to a tooth, but at the same time there was use of polymeric substances at less than 1.5 m distance from the TVOCs detector, which could add noise to the measurements. After the completion of polymeric use, TVOCs concentration decreased sharply until the second Er:YAG laser’s application. During the rest of the laser’s activity, TVOCs levels remained very high (two-fold greater than background values), indicating the laser’s important contribution to total TVOCs concentration. Small fluctuations are attributed to non-continuous laser application as well as the rest of the dental work taking place in the room.

On D16 and D18, the clinic’s windows were open, leading to much lower TVOCs’ concentration levels (Figure 6 and Figure 7), all within recommended levels, for the 1-h time frame of recordings.

Results revealed a small increase (of TVOCs) associated with every application of the Er:YAG laser, as shown in the corresponding figures. The influence of other dental work’s effects on air quality should be taken into account. When windows were open, values were lower and concentrations returned to background levels rapidly, but the laser’s influence was still detectable.

## 4. Discussion

Nowadays, laser devices are being more frequently adopted in Dentistry, for a wide array of applications: From Endodontics [25,26], to Periodontics [27], to Operative Dentistry [28], to Orthodontics [29], to name a few. Even extremely hard materials such as zirconia ceramics can now be modified by new generation ultra fast lasers [30]. In cases where the applied energy is at ablative levels, a laser plume is expected.

Several studies have proven that airborne bacteria is driven from patients into the dental aerosol [5,31,32,33,34]. The bacterial count was increased during the cavity preparation [32,35] and the length of the procedure was proportional to the amount expelled [35]. These findings drew attention to the possibility of cross-infection of airborne diseases when using high speed hand pieces [36,37,38]. As mentioned in the introduction section there is a positive correlation between indoor PM2.5 (<2.5 μm) and the presence of bronchitis and asthmatic symptoms [14,15].

Several studies have shown that different dental procedures and ventilation conditions lead to different PM and total VOCs (TVOCs) concentration levels, often exceeding the safety limit values [1,8,9,10]. Air quality standards for PM and TVOCs are being regularly reviewed and adjusted to new research results. According to DIRECTIVE 2008/50/EC [39] there is yet no identifiable limit value below which PM2.5 would not pose a risk and that is why these particles should not be regulated in the same way as other air pollutants. The World Health Organization provides as limit concentration values for daily exposure 25 μg/m^3^ for PM2.5 and 50 μg/m^3^ for PM10 [40]. The Environmental Protection Agency suggests 35 μg/m^3^ for PM2.5 and 150 μg/m^3^ for PM10 as threshold values [41]. TVOC’s also have severe influence on human health and the most commonly accepted threshold safety value is considered that of 300 μg/m^3^ [42], although the general suggestion is to reduce human exposure to the least possible. In this context, reduction of both PM and TVOCs concentrations should be a priority for dental professionals in order to ensure a non-hazardous indoor air status. The Er:YAG laser theoretically has an absorption coefficient of water that is 10-fold higher than the CO_2_ irradiation and 15,000–20,000 higher than Nd:YAG lasers [43].

Thus, the application of Er:YAG laser irradiation on hard dental tissues leads to the production of a laser plume. This is a result of the dehydration of the tissue, the cavitation of irradiated water content and subsequent heating of the residual solid matter to temperatures sufficient for combustion. After that, oxygen, present in ambient air, will combine with tissue elements to form a variety of by-products, many of which are unsafe [44]. Hence, ablation of infected tissue can create susceptibility to cross-infection due to the possible presence of viable intact infectious agents in the laser plume. As early as 1987, Frietag and associates accomplished an in vivo study that stressed the danger underlying inhalation of Nd:YAG laser smoke. In the light of their results, they concluded that the muco-ciliary function of the lung was significantly depressed and that this depression was dose-dependent [45]. Around the same time, Garden and co-workers reported viable DNA of papilloma virus in the laser plume produced after irradiating verrucae [46], while Baggish and his colleagues traced the presence of HIV pro-viral DNA in laser smoke, originating from concentrated tissue culture pellets infected with HIV [47]. Both studies used a carbon dioxide laser. In 1991, a report confirmed the transmission of human papillomavirus DNA through Nd:YAG laser smoke [48]. Also McKinley and Ludow reported that the smoke produced after Argon laser irradiation of root canals of extracted teeth inoculated with *E. coli* was positive for growth of the bacterium used [18].

Although there are papers describing the change in air quality within everyday dental procedures, the two experiments described herein demonstrated Er:YAG lasers’ effects. In the first experiment, PM10 and PM2.5 concentrations were increased by approximately 10 and 15 times, respectively. Small room dimensions and no ventilation maintained particle concentrations at very high levels for a long period of time. The most worrying aspect of these findings (with respect to potential health risks) was the fact that combustion of organic matter caused an extremely high concentration of fine particles, which are more respiratory.

From the second experiment it can be concluded that neither of the measured particles concentrations exceeded the recommended indoor limit values while windows were open, although laser influence was still detectable. Therefore, the contribution of physical ventilation in keeping PM concentration at lower levels is very important regardless of the dental procedures taking place in the clinic.

The fact that various dental procedures were also taking place during these experiments and measurements decreases the accuracy in the quantification of the Er:YAG laser’s effects, although it should not be considered neglectable. In a future project, and in order to increase the accuracy of the recordings, for the actual impact of laser-assisted dental tissue removal in a big setting environment, repeating the experiment in conditions of isolation from the rest of the clinical activity would be interesting.

Regarding the clinical use of lasers, Bahn considered some dental laser safety requirements that included the use of high-volume suction to capture laser smoke, especially if a CO_2_ laser is used [49].

Another in vivo study, examining laser plumes produced by carbon dioxide lasers during laparoscopic treatment for endometriosis and/or adhesion, demonstrated that the use of a smoke evacuator system with a high-efficiency multistage filter during plume generating laser vaporization procedures was useful. In addition, a significant portion of the particles in the laser plume were in the size range of 0.5–5.0 microns. These particles are too small to be effectively filtered by the currently available surgical masks [50]. Studies evaluating the potency of dental suction units in eliminating smoke plumes are needed. Also, rubber dam isolation in clinical practice has been shown to contribute to reduced air-borne contamination [51], but its influence on the TVOCs and Total PMs, along with high-vacuum suction, remains to be evaluated.

Recently, due to the rapid spread of COVID-19, air cleaning systems (ACS) that filtrate the air in dental offices are recommended because they may reduce aerosol contamination during cavity preparation [52]. Most of them include high-efficiency particle absorbing (HEPA) filters, which have been used in industry for many decades [53]. Those devices are basic for air purifying procedures and reduce indoor PM2.5 [54]. The use of these appliances could alter the results of this study supposing that the produced laser smoke might be partially absorbed flattening the PM and TVOCs curves.

Although these two aforementioned herein experiments took place before the outspread of the COVID-19 pandemic, dentists nowadays are facing this additional occupational hazard. It was suggested to dentists to minimize the uses of airborne spreading devices in order to reduce the production of PM particles that can act as vectors of infectious agents among patients and dentists. Most professionals respected the advice given, by carrying out only treatments deemed non-deferrable emergencies during the lockdown period [55].

## 5. Conclusions

Within the limitations applied herein, it was found that Er:YAG laser activity in a Dental Clinic, for hard dental tissue removal, was associated with high PM and TVOCs concentration values. Both experiments yielded greater increase for PM2.5 than PM10, with both of them remaining high above safety limits, for elongated time periods after the completion of laser usage. When physical ventilation was taking place, it proved to be an important key factor in improving air quality in the aforementioned setting, as both PM and TVOC concentrations decreased significantly.

## Figures and Tables

**Table 1:** Mean Particulate Matter Concentrations during Experiment 1.

	PM10	PM2.5
Background (Days 1 and 2)	28 μg/m^3^	12 μg/m^3^
Laser Activity (Day 3)	310 μg/m^3^	275 μg/m^3^

**Table 2:** Particulate Matter Concentrations (μg/m^3^) over the duration of Experiment 2 (Daily mean values).

Background Daily Values Open Windows		Background Daily Values Closed Windows	Daily Values Laser Usage Open Windows	Daily Values Laser Usage Closed Window
PM10 (μg/m^3^)	13–17	26–50	18–22	55
PM2.5 (μg/m^3^)	8–11	27–37	14	43

## Data Availability

Data sharing not applicable. No new data were created or analyzed in this study. Data sharing is not applicable to this article.
